# Isolation, Structure Elucidation and Antimicrobial Evaluation of Natural Pentacyclic Triterpenoids and Phytochemical Investigation of Different Fractions of *Ziziphus spina-christi* (L.) Stem Bark Using LCHRMS Analysis

**DOI:** 10.3390/molecules27061805

**Published:** 2022-03-10

**Authors:** Essam N. Ads, Syed I. Hassan, Saravanan Rajendrasozhan, Mona H. Hetta, Shaza H. Aly, Mohamed A. Ali

**Affiliations:** 1 Department of Chemistry, Faculty of Science, Zagazig University, Zagazig 44519, Egypt; 2 Department of Chemistry, College of Science, Sultan Qaboos University, P.O. Box 50, Muscat P.C. 123, Oman; s.hasan@squ.edu.om; 3 Department of Chemistry, College of Sciences, University of Ha’il, Ha’il 55476, Saudi Arabia; s.rajendrasozhan@uoh.edu.sa; 4 Department of Pharmacognosy, Faculty of Pharmacy, Fayoum University, Fayoum 63514, Egypt; mhm07@fayoum.edu.eg; 5 Department of Pharmacognosy, Faculty of Pharmacy, Badr University in Cairo (BUC), Badr City 11829, Egypt; 6 School of Biotechnology, Badr University in Cairo (BUC), Badr City 11829, Egypt; mohamed.ahmed_ali@buc.edu.eg

**Keywords:** antimicrobial activity, butanol extract, betulin, betulinic acid, LC-HRMS, molecular docking, *Zizyphus spina-christi*

## Abstract

*Ziziphus spina-christi* L. (ZSC-L) is a tree with thorny branches, belongs to the family Rhamnaceae and grows in the sub-tropics. The purpose of this research is to isolate and partially purify bioactive components from the crude ethanol extract of the stem bark of ZSC-L. Besides, bioassay-guided fractionation of ZSC-L stem bark was conducted using different solvents. The solvents were reutilized to minimize the production cost and environmental harm. In addition, the antimicrobial activities of the fractions were analyzed, followed by metabolic profiling using LC-HRMS. The *n*-butanol fraction showed the highest antimicrobial efficacy, so it was subjected to further purification. For the first time, two major compounds were isolated from the stem bark of ZSC-L and identified as lupane-type pentacyclic triterpenoids betulinic acid and betulin. Both compounds were used as antibacterial and anticancer agents and considered as a green product as the extraction procedure reduced the use of hazardous chemicals. Metabolic characterization of ZSC-L and its bioactive fractions were performed using LC-HR-ESI-MS and the results revealed the dereplication of 36 compounds belonging to different chemical classes. Flavonoids and triterpenes were the most prominent metabolite classes in the different fractions. The molecular docking results were obtained by studying the interaction of betulin and betulinic acid with antimicrobial receptors (4UYM, 1IYL, 1AJ2, 6J7L, 1AD4, 2VEG) to support the in vitro results. Our study highlights that *Ziziphus spina-christi* and its phytoconstituents, especially triterpenoids, act as a promising antimicrobial candidate in pharmaceutical and clinical applications.

## 1. Introduction

*Zizyphus spina-christi* L. (ZSC-L) is known as Nabka, Christ’s thorn, and Jujube plant and is distributed throughout Upper Egypt and Sinai [1]. Traditionally, it is used as a demulcent, emollient, astringent, and natural remedy for toothaches [2]. The leaves and roots are traditionally used to treat local wounds and skin conditions, respectively [3]. Furthermore, a decoction of the bark and fresh fruits is used as a body wash to enhance wound healing, while the fruits are used to alleviate dysentery [4,5]. Previous phytochemical investigations of ZSC-L stem bark revealed the existence of alkaloids, flavonoids, sterols, tannins and triterpenoids, saponins and ZSC-L stem bark reported for its antimicrobial and cytotoxic activities [6,7].

Mahran et al. reported the isolation of novel saponin glycosides from the leaves of *n*-butanol fraction of ZSC-L, namely christinin A, B, C, and D [8]. A recent study regarding the phytochemical characterization of leaves of ZSC-L resulted in the identification of 10 dammarane-type saponins and 12 phenolic compounds [9]. Flavonoids, such as quercetin, kaempferol, and phloretin derivatives, were also identified in the methanol extract of ZSC-L fruits using HPLC/ESI-MS analysis [10]. Another study reported the identification of phenolic compounds such as coumaric acid, rutin, apigenin, quercetin, chlorogenic acid and syringic acid in the methanol extract of ZSC-L stem, whereas ferulic acid, rutin, *p*-hydroxybenzoic acid and chlorogenic acid were identified in the fruits [11].

Solvent recovery and re-use in bioprocesses have the potential to decrease the pollution and waste formation substantially. However, the solvent recovery technique is typically energy-intensive and expensive [12]. In this research, an integrated bioprocess was designed for the fermentative synthesis of protopanaxadiol (PPD) from ethanol waste recycled in the downstream extraction process, which was resulted in solvent recovery and reutilization at a low cost [12].

**Figure 1:**
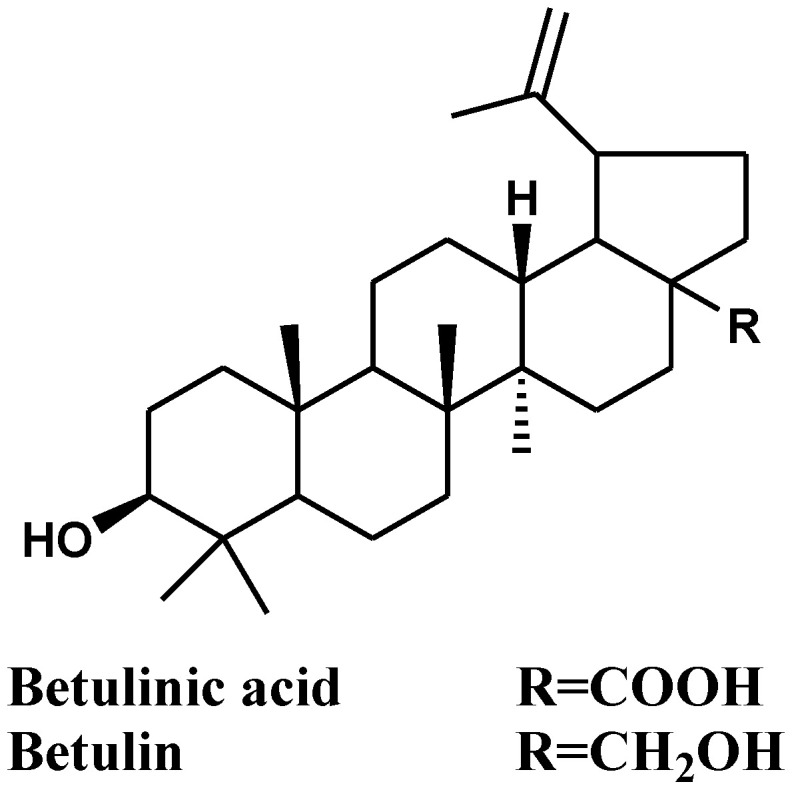
Structure of the two major compounds in the stem bark of *Z. spina-christi* L.

Betulinic acid (Figure 1), identified as 3*β*-hydroxy-lup-20(29)-en-28-oic acid, is a plant-derived pentacyclic lupane-type triterpene that is widespread among different plants: for example, *Quisqualis fructus* [13], leaves of *Vitex negundo* [14], roots of *Anemone raddeana* [15], leaves and wood of *Doliocarpus schottianus* [16], and stem bark of *Zizyphus joazeiro* [17]. A closely related compound, betulin (lup-20(29)-ene-3*β*,28-diol) (Figure 1), a natural pentacyclic triterpenoid alcohol triterpene, was isolated from the white-barked birch trees (Betula species) with the yield of 22% (dry weight) [18]. It has wide biological activities. Kim et al. reported that betulin is used as a synthetic intermediate and easily converted to betulinic acid with high yield synthetically [19]. Betulin is characterized by poor water solubility that restricts its biological activity. A recent report by Myszka et al. overcome this problem through synthesis and by testing three different d-glycosaminoside derivatives of betulin in vitro. The structure of betulin was modified by the addition of 2-amino-2-deoxy-d-gluco- and -d-galactopyranosyl groups to the C-3 position. The three new derivatives revealed potent antimicrobial activity and cytotoxicity with IC_50_ values range from 1.74 to 89.44 μM against MCF-7 breast cancer cells [20].

Previous reports revealed the biological importance of betulin as it showed adaptogenic, antioxidant, cytotoxic, anti-inflammatory, immune-modulator and hypolipemic activities [21,22,23]. Also, a combination of betulinic acid with anticancer drugs showed induction of apoptosis, caspases and inhibition of the survival of clonogenic tumor cells [24]. Betulinic acid exerts a plethora of pharmacological properties, especially as anti-inflammatory, antibacterial, antiviral agents, in addition to its antidiabetic, antimalarial, anti-HIV and antitumor properties [25].

The purpose of this study was to explore the antimicrobial activity of different fractions of *Z. spina-christi* (L.) stem bark, followed by bioassay-guided fractionation and isolation of the major bioactive compounds. Moreover, this study explores the metabolic pattern of different fractions of ZSC-L stem bark using LCHRMS.

## 2. Results and Discussion

### 2.1. Antimicrobial Activity of Different Extracts

Mean zone of inhibition in mm ± standard deviation beyond well diameter (6 mm) produced on a range of environmental and clinically pathogenic microorganisms using different crude extracts. Results are depicted in the following (Table 1) that showed *n*-butanol as the most active extract against all the microorganisms, while the diethyl extract showed no activity against any microorganism. Previous study by Haque et al. revealed that semisynthetic betulin derivatives were screened against five bacterial strains, *Enterobacter aerogenes*, *Escherichia coli*, *Enterococcus faecalis*, *Pseudomonas aeruginosa*, *Staphylococcus aureus* and a fungal strain *Candida albicans*, using broth microdilution assays. Primary antimicrobial screening at 50 µM concentration led to the identification of five compounds showing antimicrobial properties (inhibition of growth by >70% against one or more microbial strains). According to the dose-response results, 28-*O*-(*N*-acetylanthraniloyl) betulin was the most active, showing MIC values 90 of 6.25 µM against two Gram-positive bacteria, *E. faecalis* and *S. aureus* [26]. A review article by Yogeeswari et al. reported a survey of the literature dealing with betulinic acid-related biological properties that has appeared from the 1990s to the beginning of 2003. A broad range of medical and pharmaceutical disciplines are covered, including a brief introduction about discovery, phytochemical aspects, organic synthesis, anti-HIV and cytotoxic mechanisms of action. Various structural modifications were carried out and their biological and pharmacokinetic profiles are also incorporated. Betulinic acid has been shown to exhibit a variety of biological activities including inhibition of human immunodeficiency virus (HIV), antibacterial, antimalarial, anti-inflammatory, anthelmintic and antioxidant properties [22].

The *n*-Butanol fraction showed the highest antimicrobial efficacy, so it was subjected to separation using silica column chromatography. The extract was fractionated by column silica chromatography using gradient elution to obtain forty-one fractions (F1–F41). Fractions 2 and 3 eluted with chloroform-ethyl acetate (80:2 *v*/*v*) to yield two lupane-type pentacyclic triterpenoids betulinic acid (16, 20.4 mg) and betulin (23, 16.7 mg).

### 2.2. Characterization of the Isolated Compounds

The isolated pure compounds were subjected to structural characterization using melting point range, TLC and spectroscopic techniques (UV, IR, Mass and NMR). The results revealed that the structure of the isolated compounds are betulinic acid (16) and betulin (23) as compared with previous reports [28,29,30,31]. Fractions eluted from ethyl acetate-*n*-hexane (1:2) showed single spots on TLC. The R*_f_* value for this fraction was found to be 0.470, which is like the R*_f_* value of betulin in the same solvent system (0.471). Similarly, betulinic acid (16) was also eluted using the same solvent system and the R*_f_* value (0.531) was as compared with the reported data [29,32]. Results are presented in the following (Table 2). The spectral data are available in the Supplementary Material (Figures S1–S6). 

Betulinic acid (16): IR (neat, cm^−1^) 3464 (br), 2943, 2870, 1643, 1685, 1188, 883, 1454. MS: *m/z* M^+1^: 457.3305, 407(22), 353(15), 339(12), 325(100), 240(13). ^1^H NMR (CDCl_3_, 500 MHz): *δ* (ppm): 4.76 (1H, *br s*, H-29a), 4.63 (1H, *br s*, H-29b), 3.2 (1H, *m*, H-3), 1.7 (3H, *s*, H-30) 0.99 (3H, *s*), 0.98 (3H, *s*), 0.95 (3H, *s*), 0.84 (3H, *s*) and 0.77 (3H, *s*). ^13^C NMR (CDCl_3_, 125 MHz): *δ* (ppm): 38.9 (C-1), 29.7 (C-2), 79.0 (C-3), 38.7 (C-4), 55.4 (C-5), 18.3 (C-6), 34.3 (C-7), 40.7 (C-8), 50.5 (C-9), 37.2 (C-10), 20.9 (C-11), 25.7 (C-12), 38.4 (C-13), 42.4 (C-14), 30.6 (C-15), 32.2 (C-16), 56.3 (C-17), 46.9 (C-18), 49.3 (C-19), 142.6 (C-20), 29.7 (C-21), 34.3 (C-22), 27.9 (C-23), 15.3 (C-24), 16.1 (C-25), 16.0 (C-26), 14.7 (C-27), 178.6 (C-28), 109.7 (C-29), 19.4 (C-30).

Betulin (23): IR (neat, cm^−1^) 3421 (*br*), 2927, 2820, 1648, 1454, 1377, 1184, 1045, 886, 1454. MS: *m/z* M^+^: 442.3799, 393(12), 279(23), 203(100), 189 (93), ^1^H NMR (CDCl_3_, 500 MHz): *δ* (ppm): 4.70 (1H, *br s*, H-29a), 4.58 (1H, *br s*, H-29b), 3.79 (1H, *d*, *J* = 10.8 Hz, H-28b), 3.33 (1H, *d*, *J* = 10.8 Hz, H-28a), 3.18 (1H, *dd*, *J* = 9.8, 5.3 Hz, H-3*α*), 1.67 (3H, *s*, H- 30), 0.99 (3H, *s*, H-27), 0.97 (3H, *s*, H-26), 0.96 (3H, *s*, H-23), 0.80 (3H, *s*, H-25), 0.75 (3H, *s*, H-24). ^13^C NMR (CDCl_3_, 125 MHz): *δ* (ppm): 38.9 (C-1), 27.5 (C-2), 79.3 (C-3), 38.8 (C-4), 55.4 (C-5), 18.4 (C-6), 34.1 (C-7), 41.0 (C-8), 50.5 (C-9), 37.4 (C-10), 20.9 (C-11), 25.3 (C-12), 37.2 (C-13), 42.8 (C-14), 27.1 (C-15), 29.2 (C-16), 47.9 (C-17), 49.9 (C-18), 48.8 (C-19), 150.9 (C-20), 29.8 (C-21), 34.1 (C-22), 28.1 (C-23), 15.4 (C-24), 16.2 (C-25), 16.1 (C-26), 14.8 (C-27), 60.6 (C-28), 109.8 (C-29), 19.2 (C-30).

### 2.3. HPLC-ESI-MS/MS Analysis of Different Extracts of Zizyphus spina-christi L. Stem Bark

**Figure 2:**
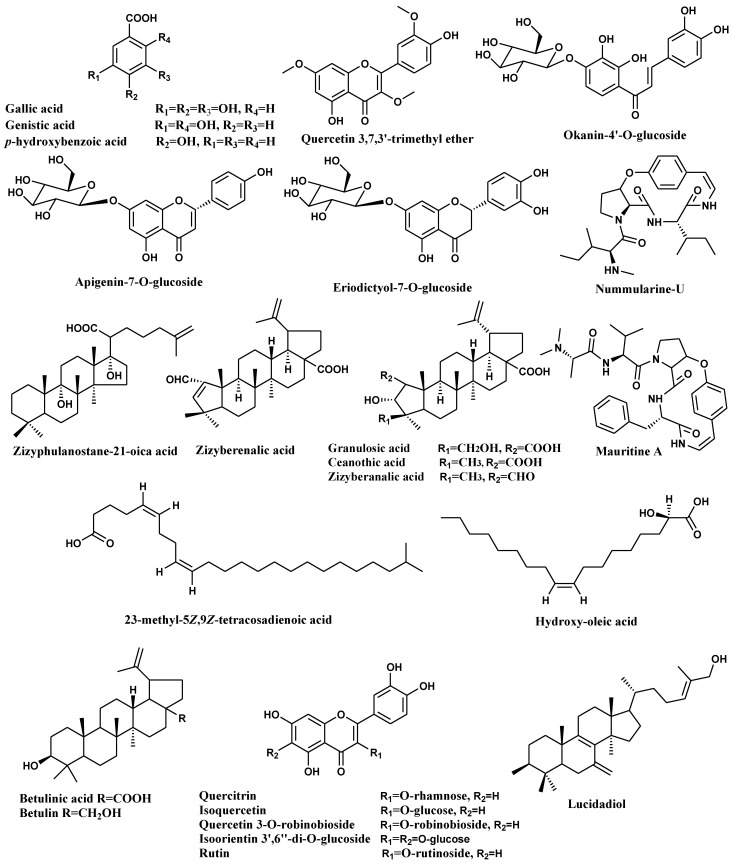
Major compounds identified in *Z. spina-christi* L. stem bark different fractions (total ethanol extract, diethyl ether, *n*-butanol) using HPLC-ESI-MS.

Previous research reported the identification of various bioactive metabolites from *Z. spina-christi* L. [7,10,33,34]. In this study, LC-HR-MS analysis was used to identify metabolites from various fractions of ZSC-L. The results revealed the presence and identification of 36 compounds from different fractions belonging to different phytochemical classes as organic acids, alkaloids hydrocarbons, triterpenes, and fatty acids, where flavonoids and triterpenes were recognized as the most prevalent class of compounds in different fractions. The tentatively identified secondary metabolites are listed in Table 3. Compounds are illustrated in Figure 2. This is the first comprehensive metabolites profiling of ZSC-L. using HPLC-ESI-MS. The metabolite profile of ZSC-L. indicates the presence of 11 flavonoids tentatively identified as quercetin 3,7,3′-trimethyl ether (4), eriodictyol-7-*O*-glucoside (7), apigenin-7-*O*-glucoside (8), okanin-4′-*O*-glucoside (15), quercitrin (17), isoquercetin (19), kaempferol-3-*O*-α-l-arabinoside (29), luteolin 7,3′-diglucoside (30), isoorientin 3′,6′′-di-*O*-glucoside (34), quercetin 3-*O*-robinobioside (35), rutin (36). Among these flavonoids, (17), (19), (34), (35) and (36) were previously isolated from ZSC-L [10,34]. Besides, eight triterpenes were suggested with molecular formula C_30_H_46_O_6_, C_30_ H_50_ O_3_, C_30_H_46_O_5_, C_30_H_46_O_4_, C_30_H_48_O_3_, C_32_H_52_O_2_, C_30_H_50_O_2_ and C_30_H_44_O_3_; identified as granulosic acid (9), zizyphulanostane-21-oica acid (12), ceanothic acid (13), zizyberanalic acid (14), betulinic acid (16), lupeol acetate (20), betulin (23), and zizyberenalic acid (26).

Additionally, fatty acid compounds identified and characterized as hydroxy-oleic acid (5), 23-methyl-5*Z*,9*Z*-tetracosadienoic acid (11), trihydroxy-oleic acid (25), *n*-hexadecanoic acid (27), hexadecanoic acid, ethyl ester (28), docosanedioic acid (31), octadecanoic acid, ethyl ester (33) based on their *m/z* 298.036, 378.3493, 330.2762, 256.2388, 284.2933, 370.307, 312.5, respectively, and in accordance with the molecular formulas C_18_H_34_O_3_, C_25_H_46_O_2_,C_18_H_34_O_5_, C_16_ H_32_O_2_, C_18_H_36_O_2_,C_22_H_42_O_4_, C_20_H_40_O_2_, respectively. A recent study conducted on the lipophilic fraction of different parts of *Z. lotus* revealed the identification of 99 compounds by GC/MS analysis, where the root bark showed the highest percentage of pentacyclic triterpenoids, especially betulinic acid, while the leaves and seeds were rich in unsaturated fatty acids [35].

The peak with *m/z* 470.3367, with a molecular formula C_26_H_39_N_4_O_4_, was identified as the cyclopeptide alkaloid, nummularine-U (10), which was previously isolated from stem bark of *Z. spina-christi* [33]. Also, another cyclopeptide alkaloid was characterized as mauritine A (6) and teleocidin B-1 (21) that were reported previously in *Zizyphus mauritiana* [36]. Four phenolic acids were characterized as gallic acid (1), genistic acid (2), *p*-hydroxybenzoic acid (3) and sinapoyl malate (24). Moreover, two sterol and one hydrocarbon were identified as stigmast-7-en-3-ol (18), lucidadiol (22), and tetracosane (32), respectively. Cadi et al. reported the polyphenolic components of *Z. lotus* fruits through PLC-DAD-ESI/MS analysis, where the most abundant compound in the ethyl acetate extract was *p*-hydroxybenzoic acid and in the methanol extract quercetin 3-*O*-rhamnoside-7-*O*-glucoside was the major component [37]. By comparing our results with the previously mentioned literature regarding the different species and different parts of *Zizyphus***;** the different fractions of *Z. spina-chritis* L. stem bark are a rich source of pentacyclic triterpenoids, fatty acids and polyphenolic compounds. 

### 2.4. In Silico Molecular Docking

**Figure 3:**
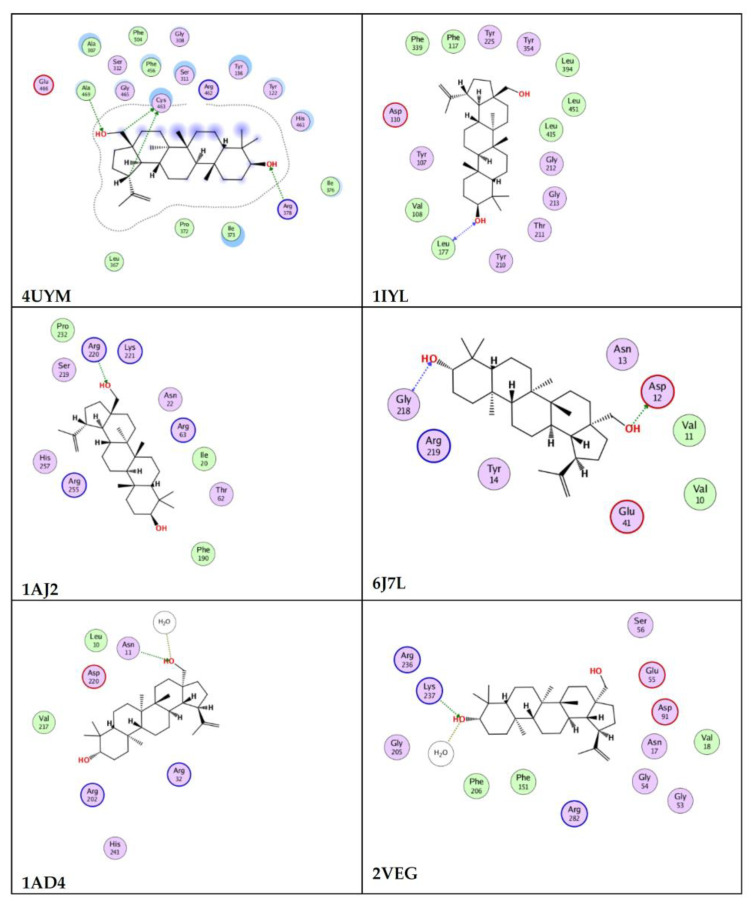
Interaction of 4UYM, 1IYL, 1AJ2, 6J7L, 1AD4, and 2VEG proteins with ligand betulin.

**Figure 4:**
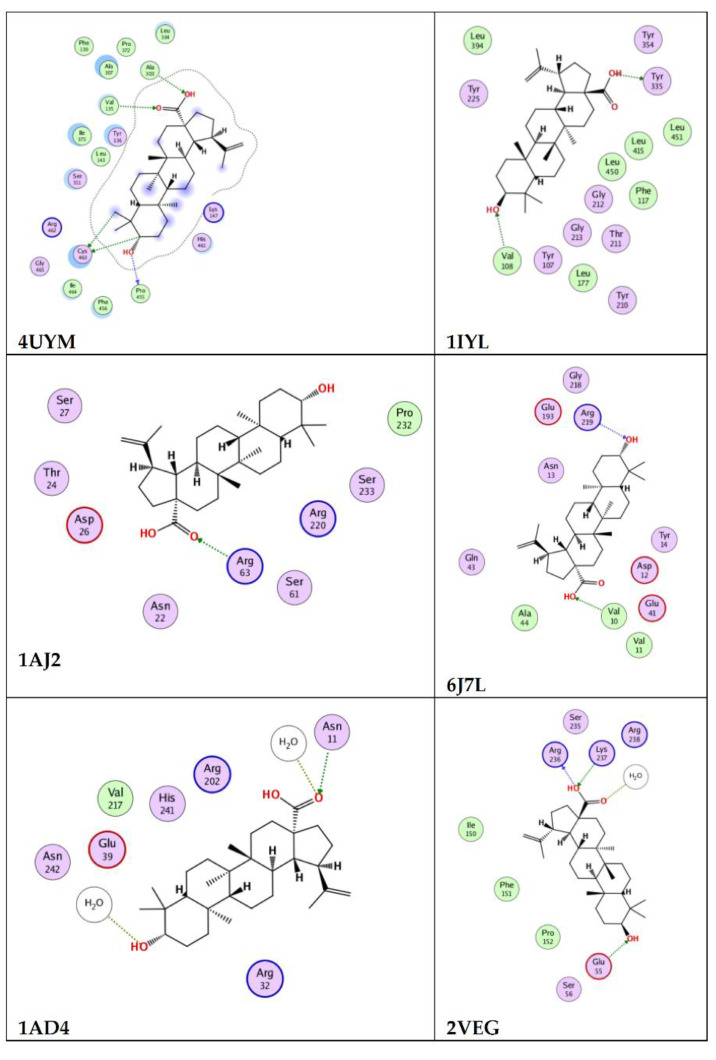
Interaction of 4UYM, 1IYL, 1AJ2, 6J7L, 1AD4, and 2VEG proteins with ligand betulinic acid.

Giving account to the retrieved antimicrobial activity, an in silico molecular docking study was conducted on betulin and betulinic acid to study their potential mechanism (Table 4). In this context, among the six potential microbial enzymes used in the docking analysis, betulinic acid showed higher antifungal activity against *A. Fumigatus* by interacting with the 4UYM protein (sterol 14-alpha demethylase) with docking score of −12.3. and betulin showed strong binding affinity with 1IYL protein (*C. albicans* N-myristoyltransferase), achieving a docking score of −13.5. Betulin and betulinic acid interact with 1AD4 protein (dihydropteroate synthetase) via hydrogen bonding to exhibit the antibacterial activity against *S. aureus*, achieving docking scores of −7.4 and −8.8, respectively. With the 2VEG protein (dihydropteroate synthase), betulinic acid and betulin interacted via hydrogen bonding with binding score of −9.4 and −10.8, respectively, to exhibit antibacterial activity against *S. pneumonia*. Betulin and betulinic acid showed good binding affinity with 1AJ2 protein (dihydropteroate synthase) with docking scores of −11.5 and −9.2, respectively, to exhibit antibacterial activity against *E. coli*. The ligand–protein interaction behaviors were estimated based on the docking score function. In general, both compounds achieved acceptable binding affinities with all the targets as well as good interaction pattern. The results suggest that betulin and betulinic acid may have different mechanisms as antimicrobial agents. Figure 3 and Figure 4 show the feasible binding geometries of betulin and betulinic acid with the target proteins.

Betulin has been reported for its molecular interaction with the target DNA gyrase A of *S. aureus* by computational docking tools. Results revealed its strong affinity toward the DNA Gyrase A with docking score −9.23 [59]. Another report by Rajkumari et al. revealed the efficacy of betulin and betulinic acid in inhibition of quorum sensing (QS)-mediated virulence factors in *P. aeruginosa*, and they serve as potent competitive inhibitors through restricting the binding of the natural ligands to the QS receptors, LasR and RhlR [60].

## 3. Materials and Methods

### 3.1. Chemicals

All solvents used for this study obtained from Sigma-Aldrich, Fisher Scientific, Scharlau Spain and VWR BDH Prolabo chemical (analytical grade).

### 3.2. Plant Material

Fresh bark of the *Z. spina-christi* was collected from the Hail area of Saudi Arabia using GPS coordinates (27.48472222, 41.69555556), (27.51416667, 41.70027778), (27.53833333, 41.69500000) and (26.00583333, 40.47222222). The taxonomic authentication was performed kindly by Dr. Sherif Sayed Sharawy, professor of taxonomy, University of Hail. A voucher specimen of the plant material was deposited in the Herbarium of the Biology department, University of Hail. The bark was air-dried, split into pieces, and stored in a dry area for future investigation.

### 3.3. Preparation of the Plant Extract

The stem bark of *Z. spina-christi* L (5.2 kg) was air-dried and extracted with absolute ethanol three times (3 × 20 L). The ethanol extract was filtered and concentrated under reduced pressure to provide 119.59 g of total extract. It was then fractionated using multiple solvents, including diethyl ether (2.5 L × 2), chloroform (2.5 L × 2), ethyl acetate (2.5 L × 2), and *n*-butanol (2.5 L × 2). Separately, the solvents were evaporated under reduced pressure to obtain 41.20 g of diethyl ether residue, 8.45 g of chloroform extract, 7.20 g of ethyl acetate extract, and 4.18 g of *n*-butanol extract, individually. The antibacterial activity of these fractions was investigated.

### 3.4. Antimicrobial Activity

The antimicrobial activity was performed against fungi (*Aspergillus fumigatus*: RCMB 02568, *Candida albicans*: RCMB 05036), Gram-positive (*Streptococcus pneumoniae*: RCMB 010010, *Staphylococcus aureus* RCMB 010028), and Gram-negative bacteria (*Pseudomonas aeruginosa*: RCMB 010043, *Escherichia coli*: RCMB 010052) by using agar well diffusion technique, as previously described in [6,27]. Test organisms were obtained from the Regional Center for Mycology and Biotechnology (RCMB), Cairo, Egypt. Data are expressed in the form of mean ± SD.

### 3.5. Separation and Purification of the Plant Metabolites from n-Butanol Fraction

The *n*-butanol extract was subjected to column chromatography on silica gel and eluted using *n*-hexane (100%), chloroform (100%), followed by gradient elution of chloroform: ethyl acetate from 100% to 0%, followed by ethyl acetate: methanol from 100% to 0%, followed by methanol-acetonitrile (75:25 *v*/*v*; 50:50 *v*/*v* and 25:75 *v*/*v*), ended by 3 volumes of methanol-acetonitrile-formic acid (5:4:1 *v*/*v*/*v*), which ultimately yielded forty-one fractions (F1–F41). Fractions collected thereof were then analyzed using TLC with different mobile phases, and similar fractions were combined together and concentrated under reduced pressure. The CAMMAG^®^ LINOMAT 5 application system was used to automatically spot fractions on TLC plates, and the development operations were carried out using two solvent systems: chloroform: methanol (9:1; *v*/*v*) or toluene: ethyl acetate: formic acid (TEF, 7: 5: 1; *v*/*v*/*v*). After drying at room temperature, the purity of fractions was determined using a CAMMAG^®^ TLC scanning device at Al-Azhar University’s Regional Center for Mycology and Biotechnology (RCMB). Fractions 2 and 3 eluted with chloroform-ethyl acetate (80:2 *v*/*v*) were then purified by preparative TLC plates using chloroform-ethyl acetate (90:10) as developer to afford two lupane-type pentacyclic triterpenoids betulinic acid (16, 20.4 mg) and betulin (23, 16.7 mg). The pure compounds were recognized based on their UV absorbance at 254 and 365 nm or visible light appearance and R_f_ value. Also, the TLC plates were visualized by vanillin-sulphuric acid spray reagent and heating them for 10 min at 120 °C.

### 3.6. LC-HR/ESI-MS Analysis of Different Extracts of Z. spina-christi L. Stem Bark

Tentative metabolite assignments were obtained by comparing mass spectral data of the identified compounds in both negative and positive ionization modes with previously reported data, as well as data from online public databases to which references were added Table 3.

The Q-TOF-LC/MS system, 6530 (Agilent Technologies) equipped with an autosampler (G7129A), a Quat. Pump (G7104C) and a column comp (G7116A) were used for chromatographic separation. The injection volume was 8 µL. The analytes were separated on a Zorbax RP-18 column from Agilent Technologies (dimensions: 150 mm × 3 mm, dp = 2.7 µm) in a flow rate of 0.3 mL/min. The mobile phase consisted of a combination of solvent A Water (0.1 formic acid) and solvent B (acetonitrile + 0.1% formic acid). The gradient elution was as follows: t = 0 min, 3% B; t = 15 min, 10% B; t = 40 min, 20% B; t = 70 min, 40% B; t = 90 min, 60% B; t = 110 min, 80% B; t = 120 min, 90% B and t = 135 min, 100% B. Mass spectra were simultaneously acquired using ESI in positive ionization mode with a capillary voltage of 4000 V. The mass spectra were recorded in the *m/z* range of 40 to 1500 *m/z*. The gas temperature and drying gas flow were 350 °C and 10 L/min, respectively.

### 3.7. In Silico Molecular Docking Studies

Six potential targets for betulin and betulinic acid were downloaded from the protein data bank (www.pdb.org) (accessed on 29 January 2022). The following IDs were used, 4UYM, 1IYL, 1AJ2, 6J7L, 1AD4, 2VEG [61,62,63,64,65,66] for sterol 14-alpha demethylase (CYP51B) from a pathogenic *Aspergillus fumigatus*; *Candida albicans* N-myristoyltransferase, *E. coli* dihydropteroate synthase, *Pseudomonas aeruginosa* Earp, *Staphylococcus aureus*, dihydropteroate synthase and Dihydropteroate synthase from *Streptococcus pneumonia*, respectively. All docking simulations were conducted using MOE software [67]. The receptor and the ligand were prepared using the standard structure optimization protocol of the software. The active site was set as where the co-crystalized ligand was bound in each corresponding target. The docking was performed using a molecular database of betulin and betulinic acid and following the induced fit protocol of MOE software [68]. The London dG and triangular matcher algorithms were used as scoring and placement methods, respectively. Each co-crystalized ligand was redocked in the vicinity of each target and the RMSD between the re-docked pose and co-crystalized ligand was calculated and used to confirm the docking validity. MOE 2019 interface was used to visualize and analyze the docking results as well as produce 2D interaction images. The docking validation data are available in the Supplementary Material (Table S1).

## 4. Conclusions

In the present work, different fractions of *Z. spina-christi* L. exhibited a varying degree of antimicrobial activity. Besides, LC-HR-MS analysis was used to identify metabolites of different fractions of *Z. spina-christi* L. The results revealed the presence and identification of 36 phytochemical compounds and biological studies carried out on the stem bark of *Z. spina-christi* L. Phytochemical investigations led to the isolation of two pure compounds, betulinic acid (C_30_H_48_O_3_) and betulin (C_30_H_50_O_2_). The structure of these compounds was determined by IR spectroscopy, mass spectroscopy, ^1^H and ^13^C NMR and confirmed by comparing with the previously reported values. The molecular docking studies on betulinic acid and betulin against enzymes in various microorganism revealed the potential binding affinity to the site of the appropriate targets. The *n*-butanol fractions of *Z. spina-christi* L. have potent antimicrobial activity. Further investigation of the isolated metabolites is required to identify the bioactive compounds responsible for antimicrobial, antioxidant and cytotoxic effects that may have potentials in pharmaceutical and clinical applications.

## Figures and Tables

**Table 1:** Antimicrobial effects of various stem bark fractions of *Z. spina-christi* L.

Tested Microorganisms	Chloroform	*n*-Butanol	Diethyl Ether	Positive Control
Fungi	Zone of Inhibition			Amphotericin B
*Aspergillus fumigatus* (RCMB 02568)	NA	18.6 ± 0.58	NA	23.7 ± 1.20
*Candida albicans* (RCMB 05036)	NA	20.6 ± 1.20	NA	25.4 ± 0.58
Gram-positive Bacteria				Ampicillin
*Streptococcus pneumoniae* (RCMB 010010)	13.2 ± 0.63	18.2 ± 0.58	NA	23.8 ± 1.20
*Staphylococcus aureus* (RCMB 010028)	16.4 ± 1.20	20.1 ± 0.63	NA	27.4 ± 0.72
Gram-negative Bacteria				Ciprofloxacin
*Pseudomonas aeruginosa* (RCMB 010043)	15.3 ± 1.50	16.2 ± 0.58	NA	20.6 ± 1.20
*Escherichia coli* (RCMB 010052)	15.9 ± 0.63	18.4 ± 0.72	NA	23.4 ± 0.63

The test was performed using the diffusion agar technique described in [6,27]. Data are expressed in the form of mean ± SD, NA: no activity; RCMB: Regional Center for Mycology and Biotechnology Antimicrobial unit test organisms.

**Table 2:** Characterization of the Isolated Compound.

Physical Properties	Compound 16	Compound 23
Color	White	Yellowish white needles (in CHCl_3_-MeOH)
State	Crystalline Solid	Crystalline Solid
Melting Point	296–297 °C	244–246 °C
Solubility	Soluble in chloroform, ethyl acetate and isobutanol	Soluble in chloroform and ethyl acetate and isobutanol
R*_f_* Value	0.531 in ethyl acetate-*n*-hexane (1:2) & 0.92 in toluene: ethyl acetate: formic acid (T:E:F, 7:5:1; *v*/*v*/*v*)	0.47 in ethyl acetate-*n*-hexane (1:2) & 0.81 in toluene: ethyl acetate: formic acid (T:E:F, 7:5:1; *v*/*v*/*v*)

**Table 3:** The LC-HRESIMS metabolite profiling of *Zizyphus spina-chritis* L. stem bark different fractions (Total ethanol extract, diethyl ether, *n*-butanol).

No.	t_R_ (min.)	Compound Name	Molecular Formula	*m/z*	Total Extract	Diethyl Ether	*n*-Butanol	References
1	6.753	Gallic acid	C_7_H_6_O_5_	168.0130	+	+	-	[38]
2	12.493	Genistic acid	C_7_H_6_O_4_	154.0332	+	+	-	[38]
3	17.543	*p*-hydroxybenzoic acid	C_7_H_6_O_3_	136.0232	+	+	-	[38]
4	32.314	Quercetin 3,7,3′-trimethyl ether	C_18_H_16_O_7_	345.1512	+	-	-	[39]
5	40.872	Hydroxy-oleic acid	C_18_H_34_O_3_	298.0363	-	+	-	[40]
6	51.079	Mauritine A	C_32_H_41_N_5_O_5_	576.3172	+	-	-	[36]
7	53.425	Eriodictyol-7-*O*-glucoside	C_21_H_22_O_11_	450.2016	+	-	+	[41]
8	65.849	Apigenin-7-*O*-glucoside	C_21_H_20_O_10_	432.1981	-	-	+	[42]
9	74.425	Granulosic acid	C_30_H_46_O_6_	504.3203	+	-	-	[43]
10	84.917	Nummularine-U	C_26_H_39_N_4_O_4_	470.3367	+	-	-	[33]
11	87.875	23-methyl-5*Z*,9*Z*-tetracosadienoic acid	C_25_H_46_O_2_	378.3493	-	+	-	[44]
12	90.720	Zizyphulanostane-21-oica acid	C_30_H_50_O_3_	458.3386	+	-	-	[45]
13	91.314	Ceanothic acid	C_30_H_46_O_5_	487.3407	+	+	-	[46]
14	92.362	Zizyberanalic acid	C_30_H_46_O_4_	470.3292	+	+	-	[46]
15	95.412	Okanin-4′-*O*-glucoside	C_21_H_22_O_11_	451.3191	+	-	-	[47]
16	97.288	Betulinic acid	C_30_H_48_O_3_	457.3305	+	-	+	[48]
17	100.690	Quercitrin	C_28_H_48_O_4_	448.3563	+	-	-	[34]
18	100.826	Stigmast-7-en-3-ol	C_29_H_50_ O	414.3121	+	+	-	[49]
19	102.449	Isoquercetin	C_21_H_20_O_12_	464.3511	+	-	-	[10]
20	102.533	Lupeol acetate	C_32_H_52_O_2_	468.3028	-	+	-	[40]
21	109.603	Teleocidin B-1	C_28_H_41_N_3_O_2_	451.3204	+	-	-	[50]
22	111.252	Lucidadiol	C_30_H_48_O_3_	456.3594	+	-	+	[51]
23	113.057	Betulin	C_30_H_50_O_2_	442.3799	+	-	+	[52]
24	116.061	Sinapoyl malate	C_15_H_16_O_9_	340.2317	+	+	-	[53]
25	117.696	Trihydroxy-oleic acid	C_18_H_34_O_5_	330.2762	+	+	+	[50]
26	122.270	Zizyberenalic acid	C_30_H_44_O_3_	452.3086	+	-	-	[54]
27	123.091	*n*-Hexadecanoic acid	C_16_H_32_O_2_	256.2388	+	-	-	[55]
28	125.489	Hexadecanoic acid, ethyl ester	C_18_H_36_O_2_	284.2933	+	+	+	[7]
29	126.36	Kaempferol-3-*O*-α-l-arabinoside	C_20_H_18_O_10_	418.3809	+	+	+	[56]
30	128.231	Luteolin 7,3′-diglucoside	C_27_H_30_O_16_	610.1543	+	-	+	[57]
31	130.029	Docosanedioic acid	C_22_H_42_O_4_	370.3070	+	-	-	[58]
32	133.177	Tetracosane	C_24_H_50_	338.3404	+	+	+	[55]
33	134.497	Octadecanoic acid, ethyl ester	C_20_H_40_O_2_	312.3246	+	+	+	[7]
34	139.627	Isoorientin 3′,6′′-di-*O*-glucoside	C_32_H_38_O_21_	758.1921	+	-	+	[10]
35	150.535	Quercetin 3-*O*-robinobioside	C_27_H_30_O_16_	610.1823	+	-	-	[10]
36	154.874	Rutin	C_27_H_30_O_16_	610.1823	+	+	+	[10]

(+): present, (-): absent.

**Table 4:** The predicted docking scores of betulin and betulinic acid for inhibitor binding with tested proteins.

Targets Proteins	Compound	Docking Scores
*A. Fumigatus* (4UYM)	Betulin	−11.7
	Betulinic acid	−12.3
*C. Albicans* (1IYL)	Betulin	−13.5
	Betulinic acid	−12.1
*E. coli* (1AJ2)	Betulin	−11.5
	Betulinic acid	−9.2
*P. aeruginosa* (6J7L)	Betulin	−7.6
	Betulinic acid	−7.7
*S. aureus* (1AD4)	Betulin	−7.4
	Betulinic acid	−8.8
*S. pneumonia* (2VEG)	Betulin	−10.8
	Betulinic acid	−9.4

## Data Availability

Data are available upon request from the first author.

## Supplementary Materials

The following supporting information can be downloaded at: https://www.mdpi.com/article/10.3390/molecules27061805/s1, Figures S1–S6: Spectral data of the isolated compounds; Table S1. The docking validation data.

## Sample Availability

Samples of the plant material and different fractions may be available from the authors.
