# Supplementary material for: Isolation, Structure Elucidation and Antimicrobial Evaluation of Natural Pentacyclic Triterpenoids and Phytochemical Investigation of Different Fractions of Ziziphus spina-christi (L.) Stem Bark Using LCHRMS Analysis
Source: Molecules. 2022 Mar 10;27(6):1805. doi: 10.3390/molecules27061805 (PMC8951313; doi:10.3390/molecules27061805)
Supplement: Supplementary file 1 [file molecules-27-01805-s001.zip › molecules-1552619-supplementary.pdf]

Isolation and Structure Elucidation of Natural Pentacyclic Triterpenoids and Phytochemical Investigation of Different Fractions of *Ziziphus spina-christi* (L.) Stem Bark Using LCHRMS Analysis

Essam N. Ads [1,*], Syed I. Hassan [2], Saravanan Rajendrasozhan [3], Mona H. Heta [4], Shaza H. Aly [5,*] and Mohamed A. Ali [6]

[1] Department of Chemistry, Faculty of Science, Zagazig University, Zagazig 44519, Egypt; essam.adss@yahoo.com
[2] Department of Chemistry, College of Science, Sultan Qaboos University, P.O. Box 50, Muscat P.C. 123, Oman; s.hasan@squ.edu.om
[3] Department of Chemistry, College of Sciences, University of Ha'il, Ha'il 55476, Saudi Arabia; s.rajendrasozhan@uoh.edu.sa
[4] Department of Pharmacognosy, Faculty of Pharmacy, Fayoum University, Fayoum 63514, Egypt; mhm07@fayoum.edu.eg
[5] Department of Pharmacognosy, Faculty of Pharmacy, Badr University in Cairo (BUC), Badr City 11829, Egypt; shaza.husseiny@buc.edu.eg
[6] School of Biotechnology, Badr University in Cairo (BUC), Badr City 11829, Egypt; mohamed.ahmed_ali@buc.edu.eg
* Correspondence: essam.adss@yahoo.com (E.N.A.); shaza.husseiny@buc.edu.eg (S.H.A.); Tel.: +20-15-5551-2497 or +20-10-9957-6833 or +20-50-4037-366 (E.N.A.)

Supplementary material

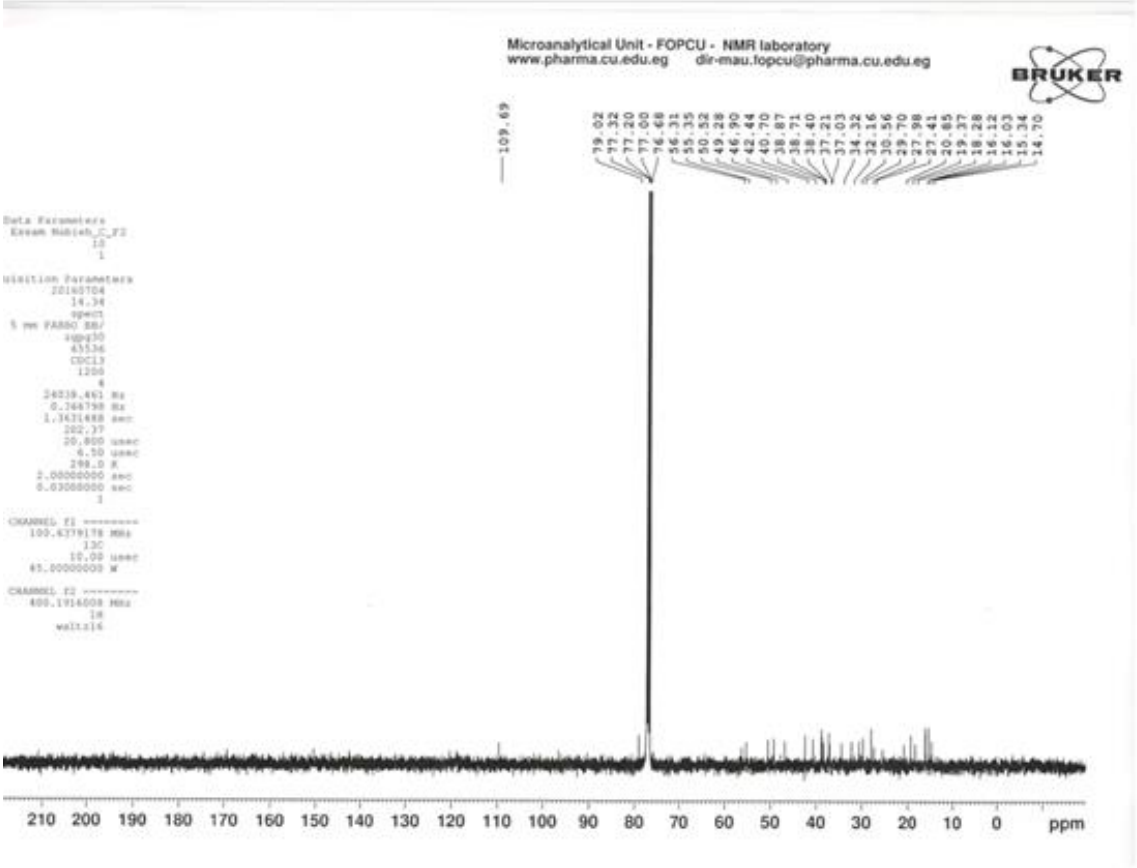

Figure S1. ^{13}C-NMR Spectra of betulinic acid

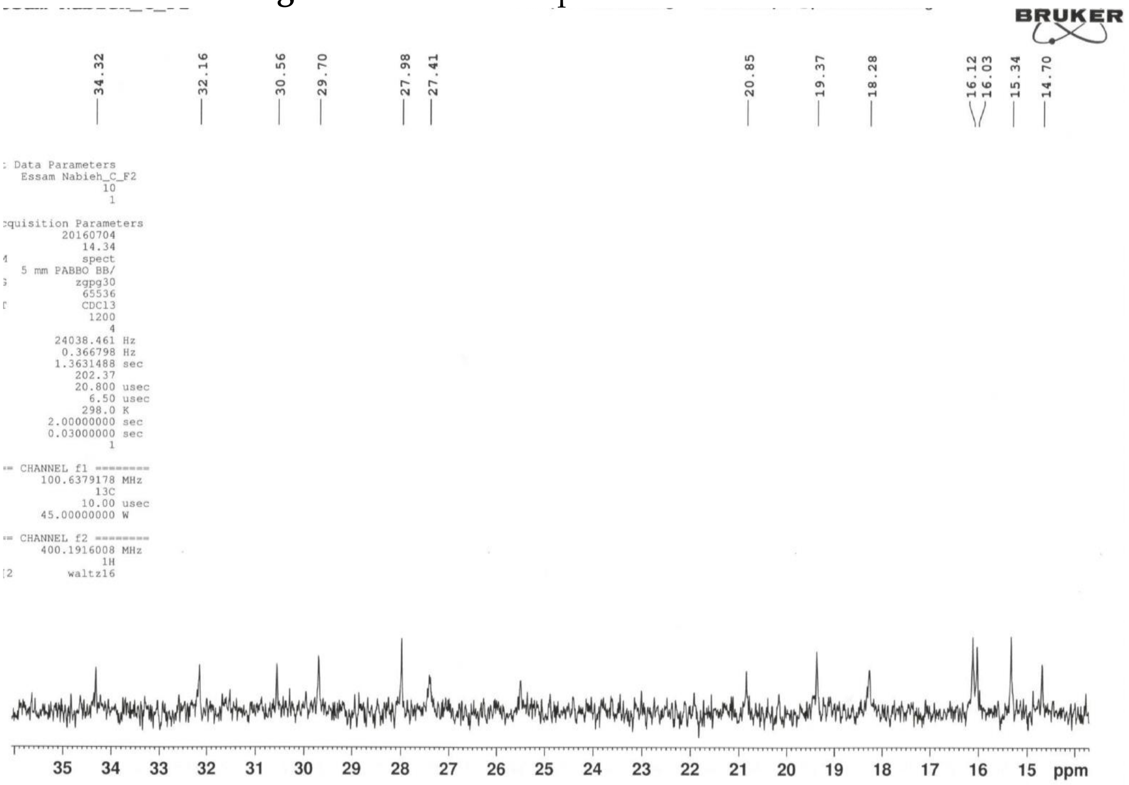

Figure S2. ^{13}C-NMR Spectra of betulinic acid (enlarged)

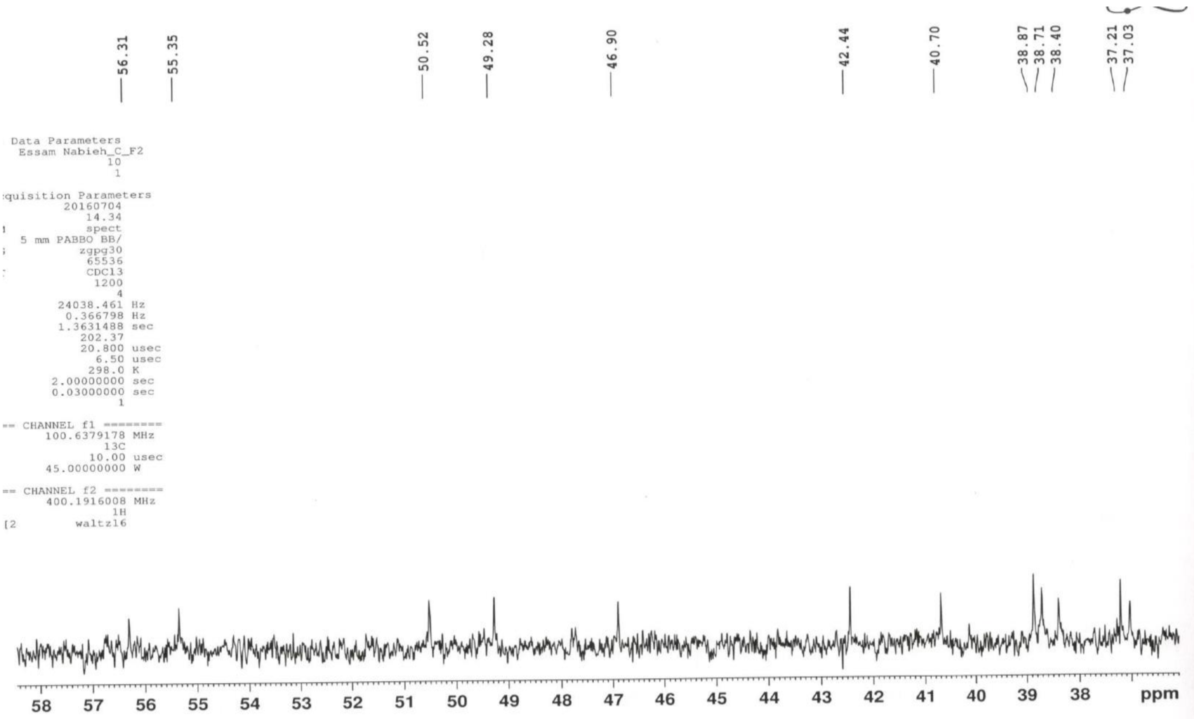

Figure S3. ^{13}C-NMR Spectra of betulinic acid (enlarged)

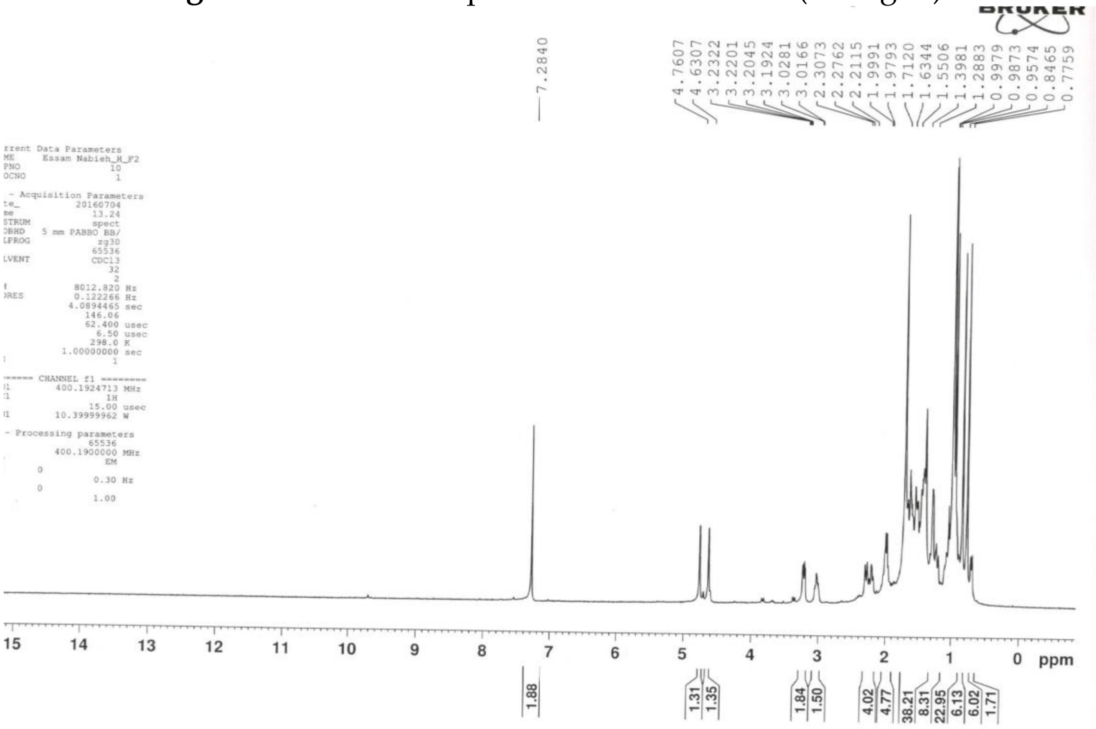

Figure S4. ^{1}H-NMR Spectra of betulinic acid

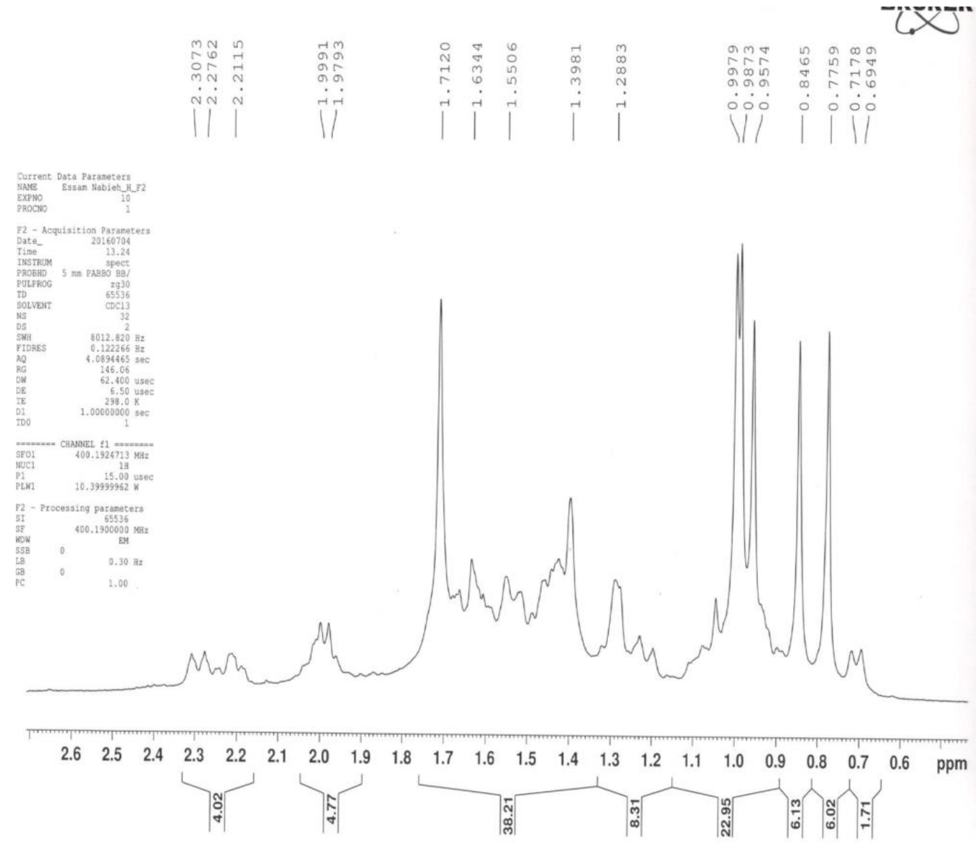

Figure S4. ¹H-NMR Spectra of betulinic acid (enlarged)

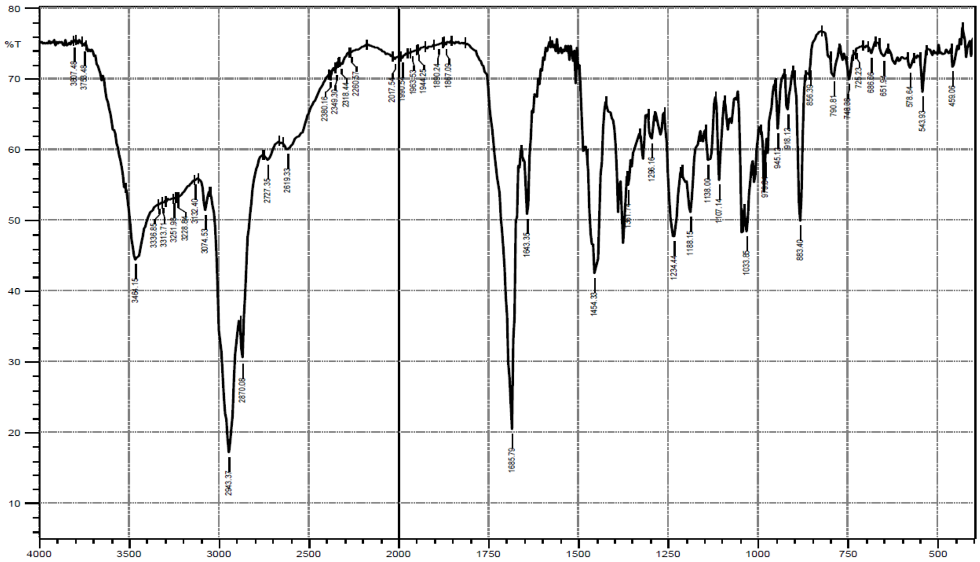

Figure S5. IR Spectrum of betulinic acid

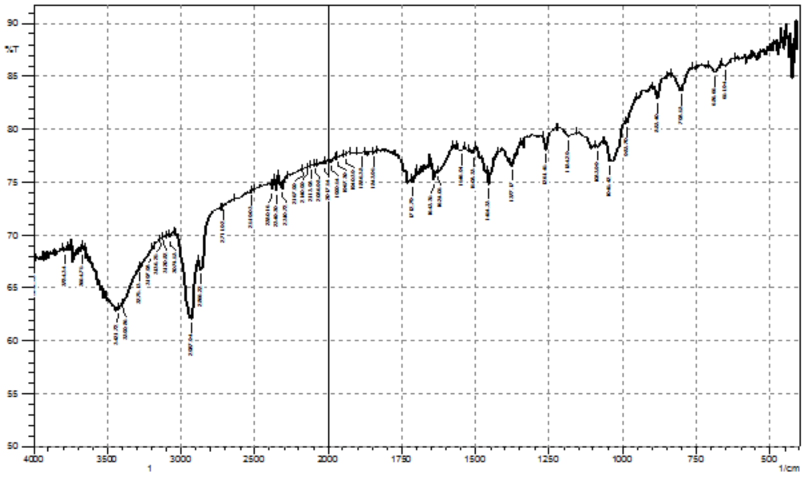

Figure S6. IR Spectrum of betuline

Table S1. Docking validation results

Protein	Control for RMSD	RMSD
Aspergillus Fumigatus	Voriconazole	1.2
C. Albicans	(1-Methyl-1H-Imidazol-2-yl)-(3-Methyl-4-{3-[(Pyridin-3-yl-methyl)-Amino]-Propoxy}-Benzofuran-2-yl)-Methanone	0.7
E.coli	[7,8-Dihydro-Pterin-6-yl Methanyl]-Phosphonophosphate	0.9
Pseudomonas aeruginosa	Thymidine-5'-Diphosphate	0.8
S. aureus	6-Hydroxymethylpterin-Diphosphate	0.6
Streptococcus pneumoniae	Pterin-6-yl-Methyl-Monophosphate	0.7
